# Tackling Multidrug Resistance in Streptococci – From Novel Biotherapeutic Strategies to Nanomedicines

**DOI:** 10.3389/fmicb.2020.579916

**Published:** 2020-10-06

**Authors:** Cinthia Alves-Barroco, Lorenzo Rivas-García, Alexandra R. Fernandes, Pedro Viana Baptista

**Affiliations:** ^1^UCIBIO, Departamento de Ciências da Vida, Faculdade de Ciências e Tecnologia, Universidade NOVA de Lisboa, Caparica, Portugal; ^2^Biomedical Research Centre, University of Granada, Granada, Spain

**Keywords:** antimicrobial resistance, biofilms, pyogenic streptococci, bacteriocins, bacteriophage, nanoparticles, nanomedicine

## Abstract

The pyogenic streptococci group includes pathogenic species for humans and other animals and has been associated with enduring morbidity and high mortality. The main reason for the treatment failure of streptococcal infections is the increased resistance to antibiotics. In recent years, infectious diseases caused by pyogenic streptococci resistant to multiple antibiotics have been raising with a significant impact to public health and veterinary industry. The rise of antibiotic-resistant streptococci has been associated to diverse mechanisms, such as efflux pumps and modifications of the antimicrobial target. Among streptococci, antibiotic resistance emerges from previously sensitive populations as result of horizontal gene transfer or chromosomal point mutations due to excessive use of antimicrobials. Streptococci strains are also recognized as biofilm producers. The increased resistance of biofilms to antibiotics among streptococci promote persistent infection, which comprise circa 80% of microbial infections in humans. Therefore, to overcome drug resistance, new strategies, including new antibacterial and antibiofilm agents, have been studied. Interestingly, the use of systems based on nanoparticles have been applied to tackle infection and reduce the emergence of drug resistance. Herein, we present a synopsis of mechanisms associated to drug resistance in (pyogenic) streptococci and discuss some innovative strategies as alternative to conventional antibiotics, such as bacteriocins, bacteriophage, and phage lysins, and metal nanoparticles. We shall provide focused discussion on the advantages and limitations of agents considering application, efficacy and safety in the context of impact to the host and evolution of bacterial resistance.

## Introduction

The pyogenic group belonging to the genus *Streptococcus* includes species are habitually part of the flora of animals (including humans) and, as such, most species are regarded as commensal, but under fitting circumstances may cause localized and systemic infections (Nobbs et al., 2009; Peters, 2017). Species of the pyogenic streptococci group include *Streptococcus pyogenes*, *Streptococcus agalactiae*, *Streptococcus dysgalactiae* subsp. *dysgalactiae* (SDSD), and *Streptococcus dysgalactiae* subsp. *equisimilis* (SDSE) which, together with *Streptococcus pneumoniae*, are the key pathogens belonging to the genus *Streptococcus* (Parks et al., 2015). For example, *S. pyogenes* is the cause of numerous severe human diseases, including septicemia and streptococcal “toxic-shock” syndrome (Isaacs and Dobson, 2016). *S. agalactiae* is the most frequent cause of sepsis and meningitis in neonates and children (Rajagopal, 2009; Melin, 2011). Considering domestic animals, *S. agalactiae* is one of the main causes of bovine mastitis (Rato et al., 2013). SDSE was primarily considered a human commensal organism but nowadays its relevance as human pathogen is on the raising, causing a similar range of diseases in humans as does *S. pyogenes* (Brandt and Spellerberg, 2009). SDSD has been considered an animal pathogen and is frequently associated with bovine mastitis (Abdelsalam et al., 2013). Human infections associated with this subspecies have been sporadically reported (Koh et al., 2009; Park et al., 2012; Jordal et al., 2015), and its role in human disease remains unclear.

In recent years, severe outbreaks of infectious diseases caused by organisms resistant to multiple antibiotics have occurred. Drug resistance is mounting globally, threatening our capability to treat common infections, resulting in persistent illness and death. It is estimated that by 2050, around 10 million human deaths per year might be attributable to antimicrobial resistance (Neill, 2014, 2016). The increase in antimicrobial resistance is more frightening derived from the considerable narrow number of new antimicrobial agents currently under development (World Health Organization, 2020). The growing of resistance in bacteria has been associated to increased consumption of antimicrobials, and improper prescribing of antimicrobials, leading to selective pressure that trigger drug resistance in exposed bacteria and, consequently, in the persistence of antibiotic resistance genes in populations of the same ecological niches, mainly as a result of horizontal gene transfer (Fair and Tor, 2014). Indeed, high-throughput sequencing and other molecular genetics tools led to a better understanding of the underlying mechanisms of horizontal gene transfer. For instance, in average, about 20% of the fully sequenced genome of *Streptococcus* consists of mobile and exogenous DNA, comprising conjugative and composite transposons, phage regions, and plasmid (Lier et al., 2015; Yamada et al., 2019). Thus, horizontal gene transfer constitutes one of the leading modes of originating gene diversity which confers new antibiotic resistance mechanisms in *Streptococcus*. These gene transfer events frequently strike in the pyogenic group, particularly in *S. pyogenes*, *S. agalactiae*, *Streptococcus canis*, SDSD, SDSE, and *Streptococcus uberis* (Haenni et al., 2010; Richards et al., 2012; Wong and Yuen, 2012; Rohde and Cleary, 2016). Too, there have been reports of an increasing incidence of multiple drug resistance (MDR) among streptococci strains, which hamper customary empirical antimicrobial therapy for these infections. Still, even though pyogenic streptococci remain susceptible to most prescribed antibiotics, treatment failure due to MDR has also been reported both in human and veterinary patients (Doumith et al., 2017; Lai et al., 2017).

The quest for effective approaches to tackle MDR bacteria has put forward several alternatives, such as competitive exclusion of pathogenic bacteria via bacteriocin, and bacteriophages (Rotello et al., 2016; Furfaro et al., 2018; Lopetuso et al., 2019). The effectiveness of some of these new approaches for therapeutics is highly variable, but positive effects have been reported in some species. Irrespective of the mechanism of action, the ways bacteria seem to be able to develop resistance to these new approaches has not received enough attention, making it more difficult to find long-term solutions. Herein, we present an overview of mechanisms of resistance to antimicrobials in pyogenic streptococci, factors that contribute to antibiotic resistance and news approach to treating infectious diseases as an alternative to antibiotics, such as bacteriocins, bacteriophage and phage lysins, and nanoparticles. We shall provide focused discussion on the advantages and limitations of agents considering application, effectiveness, resistance development, and interactions with the immune system.

## Antibiotics and Mechanisms of Resistance

**FIGURE 1:**
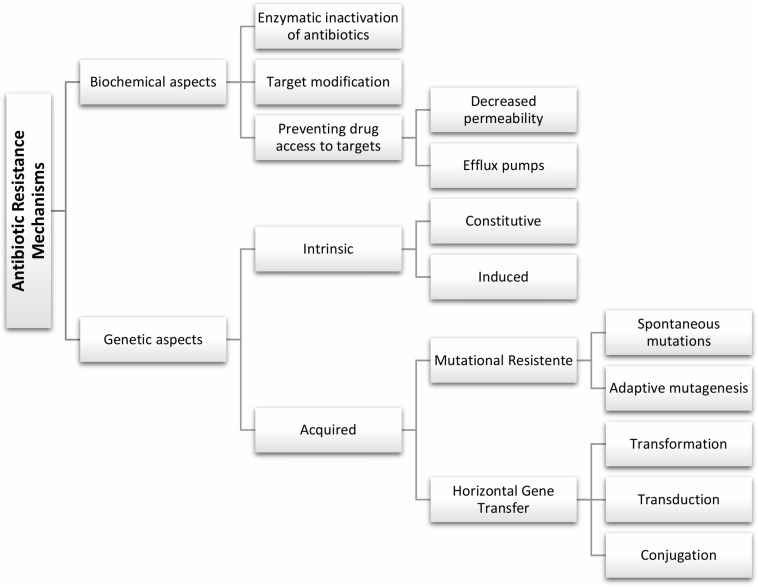
General genotypic and biochemical aspects of antibiotic resistance mechanisms.

An ideal antimicrobial ought to show high selective toxicity for bacteria with minimal adverse impact to the host (Kohanski et al., 2010). Antibacterial may be organized into four main clusters based on the mechanism of action and target in the bacterial cell – see summary in Table 1. Still, the mechanisms of resistance to antimicrobials are complex, and different mechanisms may be present in the same strain promoting a multidrug resistance phenotype, but whose main genotypic and phenotypic characteristics may be schematically grouped as shown in Figure 1. Some of these fundamental biochemical mechanisms of antimicrobial resistance include: (i) enzymatic inactivation of antibiotics, e.g., β-lactamases (Munita et al., 2016); (ii) modifications of the antimicrobial target preventing efficient binding of the antibiotic, which often results from spontaneous mutations, including genome and RNA variations (e.g., rRNA mutations associated to resistance to several antibiotics) (Malbruny et al., 2002; Gomez et al., 2017); (iii) preventing drug access to targets, for example through the reduced uptake by the cell via a decrease of outer membrane permeability in Gram-negative and/or active efflux pumps that increase clearance from within the cell (Petchiappan and Chatterji, 2017).

**TABLE 1:** Mechanisms of the main antibacterial drugs.

Mode of action	Target		Drug examples
**Antimetabolites**	Folic acid synthesis enzyme	Inhibits enzymes involved in production of dihydrofolic acid	Sulfonamides
		Inhibits the enzymes involved in the production of tetrahydrofolic acid	Trimethoprim
**Cell wall synthesis inhibitors**	Penicillin-binding proteins	Interact with PBPs and inhibit transpeptidase activity	β-lactams: penicillins (ampicillin, amoxicillin, methicillin penicillin G, penicillin V), cephalosporins (first, second, third, fourth, and fifth generations), monobactams (aztreonam), carbapenems (imipenem, meropenem, doripenem)
	Peptidoglycan subunit transport	inhibits transport of subunits across membrane	Bacitracin
	Peptidoglycan subunit	inhibits the transglycosylation and transpeptidation	Glycopeptides (vancomycin and teicoplanin)
**Nucleic acid synthesis inhibitors**	RNA	Inhibits RNA polymerase activity	Rifamycin
	DNA	Inhibits the activity of DNA gyrase and, consequently, the DNA replication	Fluoroquinolones: ciprofloxacin, levofloxacin, gatifloxacin, gemifloxacin, garenoxacin, sparfloxacin, and pefloxacin
**Protein synthesis inhibitors**	30S ribosomal subunit	Promotes mismatches between codons and anticodons, producing defective proteins that are inserted causing disrupt the cytoplasmic membrane	Aminoglycosides: streptomycin, gentamicin, neomycin, and kanamycin.
		Inhibits the interaction of tRNAs with ribosome	Tetracyclines
	50S ribosomal subunit	Blocks peptide bond formation among amino acids	Macrolides: erythromycin and azithromycin. Lincosamides: naturally produced lincomycin and semisynthetic clindamycin. Chloramphenicol.
		Inhibits the formation of the initiation complex between 50S and 30S subunits.	Oxazolidinones: including linezolid

To fully realize the propagation of antibiotic resistance, one needs to recognize the molecular mechanisms of resistance to antibiotics and to map the resistome in different ecological niches. Several studies have assessed the resistome in the environment, namely in wastewater, soil, and gut microbiota of animals (humans included) (Pehrsson, 2016; Von Wintersdorff et al., 2016). Metagenomics directly analyze DNA in a biological sample, allowing for analysis of the resistome within distinct microbial ecosystems (Von Wintersdorff et al., 2016). These studies highlight that determinants of antibiotic resistance, including those clinically relevant, are prevalent in these environments (Lehtinen et al., 2019). Sequence-based studies provide large datasets, but one limitation is that they focus on genes already known to be involved in the resistance, or (less frequently) to predict new functions based on the homology to known sequences. These genome annotation schemes will provide more and more information to complement the output of functional metagenomics, which shall result in the identification of new determinants of antibiotic resistance (Von Wintersdorff et al., 2016).

In general, bacterial drug resistance can be divided into intrinsic and acquired resistance (Reygaert, 2018). Intrinsic resistance is a naturally occurring phenomenon, which prevents antimicrobial activity and it is common to the majority of strains of a given species. The intrinsic resistance may be constitutive, i.e., independent of previous antibiotic exposure (e.g., reduced permeability of the outer membrane), or induced via the exposure to antibiotic or environmental stress (e.g., multidrug efflux pumps and biofilm formation) (Baldassarri et al., 2006; Cox and Wright, 2013). Acquired resistance is due to chromosomal point mutations or by acquisition of mobile resistance genes, in which resistant strains emerge from previously sensitive bacterial populations, customarily subsequently to exposure to the antimicrobial (Haenni et al., 2010; Enault et al., 2017).

**FIGURE 2:**
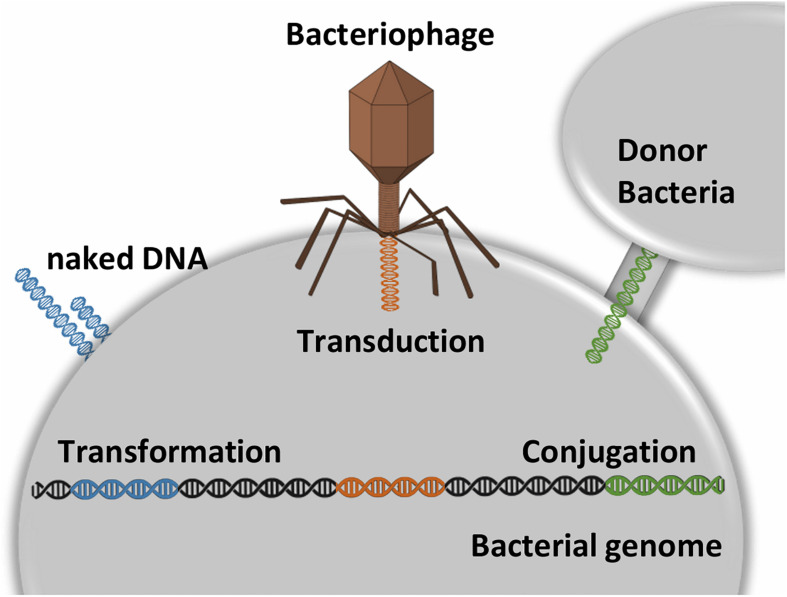
Transformation is the process by which naked DNA from the external environment is incorporated into a bacterial cell. For this process is requires the recipient cell to exhibit on its membrane special DNA binding proteins. Transduction is the process by which a phage transfers DNA from one bacterial strain to another. Conjugation is the process mediated by cell-to-cell contact that provides direct DNA transfer. Conjugative transfer systems associated with plasmids usually code the necessary proteins to DNA exchange. The plasmids are kept as extra-chromosomal genetic material by external selective pressure (e.g., presence of metal or antibiotic). Overall, these mechanisms can be followed by recombination events that allow the genetic determinants to be inserted stably into the chromosome.

The acquisition of mobile genetic elements (MGEs), such as bacteriophages, plasmids, integrative and conjugative elements, is recognized as a key point in the emergence of multidrug-resistant (MDR) strains (Lehtinen et al., 2019). The main mechanisms of DNA uptake in bacteria are conjugation, transduction, and transformation (Figure 2), which must be followed by recombination to allow stable insertion into the chromosome. These MGEs are self-transmissible elements common in bacteria. Further to genes involved in mobility, regulation, or maintenance, MGEs convey antibiotic resistance genes and virulence factors, such as exotoxins (Haenni et al., 2010). Horizontal transfer of genes (HGT) can modulate host-pathogen interactions and extending the host range. Indeed, the use of high-throughput sequencing tools allowed for a better understanding of HGT. For example, in *S. pyogenes* the lateral exchange of virulence genes, mediated by bacteriophage infection, is a very important factor in the diversification of the species. What is more, bacteriophages may convey genes that provide for selective advantage to the host, thus fostering their own dissemination (Colomer-Lluch et al., 2011; Von Wintersdorff et al., 2016).

Many determinants of resistance are frequently present on a single R plasmid (harboring several antibiotics-resistance genes), thus, multiple resistance can be shared among bacteria in single-event of conjugation (Nikaido, 2009). Many of these R plasmids contain resistance genes against the main classes of antibiotics, such as aminoglycosides, macrolides, phenicol, and tetracycline (Nikaido, 2009).

Streptococcus harbor various plasmids associated with the transfer of antibiotic resistance and virulence (Grohmann et al., 2003; Cook et al., 2013). In addition to plasmids, a wide variety of transposons have been isolated in streptococci (Brenciani et al., 2007; Fléchard and Gilot, 2014), namely Tn3-family transposons, composite and conjugative transposons. For example, Tn916, encoding *tetM* for the ribosomal protection protein TET(M), associated to independent transfer of resistance between a multitude of strains via a plasmid, including *Enterococcus faecalis*, *Staphylococcus aureus*, *S. pneumoniae*, *S. agalactiae*, and SDSD (Franke and Clewell, 1981; Haenni et al., 2010; Fléchard and Gilot, 2014; Osei Sekyere and Mensah, 2019), that act as reservoirs of functional antibiotic resistance genes.

Several mechanisms of antibiotic resistance among pyogenic streptococci have been reported, whose main mechanism of action and associated resistances shall be briefly described. Table 2 summarizes the main antibiotics used for the treatment of streptococcal infections and resistance mechanisms.

**TABLE 2:** Main mechanisms of antibiotic resistance in pyogenic streptococci.

Mechanism	Target antibiotics	Examples	Location	References
Enzyme inactivation	β-lactam	β-lactamases via hydrolisis, e.g., BL2b; TEM-1; TEM-47; TEM-71; TEM-89; TEM-95	Chromosome	Vélez et al., 2017
	Aminoglycosides	Aminoglycoside-modifying enzymes (AMEs) – APH(3′)-IIIa; ANT(6)-Ia; AAC(6′)-APH(2′′)	Chromosome and MGE, e.g., Tn4001, Tn5405 and Tn3706 (Tn4001 derivative)	Galimand et al., 1999; Prudhomme et al., 2002; Kristich et al., 2014; Cattoir, 2016; Doumith et al., 2017
	Chloramphenicol	Chloramphenicol *O*-acetyltransferase (CAT) enzyme encoded by genetic determinants *cat*(pC194); *cat*(pC221); *cat*(pSCS7); *cat*S, and *cat*Q	Plasmid and conjugative transposon Tn1545 and Tn5253-like	Schwarz et al., 2004; Woodford, 2005; Del Grosso et al., 2011; Mingoia et al., 2015
	Lincosamide	LinB/A catalyze adenylylation of antibiotics	MGE	Haenni et al., 2010; Rato et al., 2013; Osei Sekyere and Mensah, 2019
Preventing drug access to target	Tetracycline	Efflux pumps Tet(K)	Chromosomal insertion element	Rato et al., 2013; Emaneini et al., 2014; Nguyen et al., 2014
	Quinolones	Efflux pumps PmrA	Chromosome	Martinez-Garriga et al., 2007
	Macrolides	Efflux pumps Mef and Msr	MGE such as Tn917 and bacteriophage Φ m46.1.	Brenciani et al., 2004, 2007; Di Luca et al., 2010; Del Grosso et al., 2011; Rato et al., 2013; Hadjirin et al., 2014
	Tetracycline	Tet(M), Tet(O), Tet(Q), Tet(S), Tet(T), Tet(W) ribosomal protection proteins dissociate tetracycline from ribosome	MGE (Tn916 and Tn3701) and bacteriophages Φ m46.1	Brenciani et al., 2004, 2007; Liu et al., 2008; Nikaido, 2009; Di Luca et al., 2010; Nguyen et al., 2014; Da Cunha et al., 2014; Silva et al., 2015; Kim et al., 2018
Target modification	Quinolones and Fluoroquinolones	Point mutations primarily in quinolone resistance-determining regions (QRDRs), of *par*C and *gyr*A genes	Chromosome. Evidence for horizontal transfer of QRDR between streptococci has been reported.	Ferrándiz et al., 2000; Balsalobre et al., 2003; Orscheln et al., 2005; Pletz et al., 2006; Duesberg et al., 2008; Hooper and Jacoby, 2016; Pham et al., 2019
	Tetracycline; Aminoglycosides	Modification in rRNA	Chromosomal mutation	Nguyen et al., 2014; Lupien et al., 2015
	Macrolides	Modification of 23S rRNA and/or ribosomal proteins L4 and L22 determinants	Chromosomal mutation	Malbruny et al., 2002; Jalava et al., 2004
	β-lactam	Modification of penicillin-binding proteins 1A [PBP1A], PBP2B, and PBP2X	Chromosomal mutation	Kimura et al., 2008, 2013; Pillai et al., 2009; Fuursted et al., 2016; Moroi et al., 2019; Vannice et al., 2019; Musser et al., 2020
	Macrolides-Lincosamide- Streptogramin B	Modification by methylation of rRNA (*erm*-class genes)	The *erm*(B) and *erm*(TR) genes are found in the chromosome of streptococci and conjugative transposons, such as Tn916 family and Tn5397 elements	Brenciani et al., 2007, 2011; Cattoir, 2016

β-lactams, targeting the bacterial cell wall peptidoglycan, particularly enzymes linked to peptidoglycan synthesis, are one of the most prescribed antibiotics for streptococcal infections due to the broad spectrum of action (Kohanski et al., 2010; Kong et al., 2010). β-lactamases are secreted enzymes capable of destroying these antibiotics and are the most frequent cause of resistance, but not the only (Bush and Jacoby, 2010; Munita et al., 2016). In fact, pyogenic streptococci have been recognized as non-β-lactamase-producing bacteria, where resistance to β-lactams is essentially mediated by alterations to the binding site of penicillin-binding proteins (PBPs) (Vannice et al., 2019). Nevertheless, a recent study based on whole-genome sequencing revealed the presence of β-lactamases determinants of *S. uberis* and SDSD isolates bovine mastitis (Vélez et al., 2017). Still, there is a need for actional studies to assess the potential of these β-lactamases, and the role of these species as a reservoir of determinants of resistance.

Macrolides are the first choice against streptococcal infections in patients allergic to β-lactam (Kanoh and Rubin, 2010) and, clindamycin (lincosamides) has been used for the treatment of infections associated with anaerobic bacteria as an alternative to penicillin G (Greenwood and Irving, 2012). Three main mechanisms have been associated with resistance to these antibiotics: (i) target modification by methylation of rRNA (*erm* genes) or target mutations, (ii) active efflux, and (iii) enzymatic inactivation (Matsuzaki et al., 2005; Petinaki and Papagiannitsis, 2018). Although most streptococci strains remain sensitive to macrolides and lincosamides, resistance phenotypes have emerged among pyogenic streptococci (Rato et al., 2013; Cattoir, 2016; De Greef et al., 2019).

Since active electron transport is required for aminoglycoside uptake into bacteria, aminoglycosides have weak activity against anaerobic bacteria (Ramirez and Tolmasky, 2010; Krause et al., 2016). Still, low levels of resistance to aminoglycosides are observed in most *Streptococcus* spp., and high-level resistance to aminoglycosides appears to be rare. This resistance occurs due to the production of AAC(6′)-APH(2′′), APH(3′)-IIIa, and ANT(6)-Ia enzymes and has been demonstrated to be transferable by conjugation (Cattoir, 2016).

Chloramphenicol-resistant streptococci are not common even though some studies show high levels of resistance among the pyogenic group species, namely, *S. pyogenes*, *S. agalactiae*, and SDSE (Trieu-Cuot et al., 1993; Schwarz et al., 2004; Woodford, 2005). Among streptococci and other Gram-positive bacteria, the resistance to chloramphenicol is mainly mediated by Chloramphenicol *O*-acetyltransferase (CAT) enzymes encoded by plasmids or chromosomally integrated. Several CATs are shared by streptococci, staphylococci, and enterococci strains (Woodford, 2005).

Currently, fluoroquinolones (FQ) have also been put forward as a therapeutic option for the treatment of streptococcal infections (Pinho et al., 2010). However, emergence of resistance among several streptococcal species, including SDSE, *S. pyogenes*, and *S. agalactiae*, *S. pneumoniae*, and viridans group streptococci has been reported (Guerin et al., 2000; Martinez-Garriga et al., 2007; Duesberg et al., 2008; Pinho et al., 2010; Pires et al., 2010; Kimura et al., 2013; Dang et al., 2014; Arias et al., 2019). The most frequent mechanism of high-level FQ resistance is the target modification due to mutations in *parC* and *gyrA* genes that occur mainly in quinolone resistance-determining regions (QRDRs) (Hooper and Jacoby, 2016; Pham et al., 2019). Resistance to FQ can also be mediated by modifying enzymes, target-protection proteins (Pham et al., 2019) and by increased production of multidrug-resistance efflux pumps (Hooper, 2002).

Resistance to tetracyclines (TET) among streptococci strains is often found in high rates (Nakamur et al., 2011; Emaneini et al., 2014; Gherardi et al., 2014; Vélez et al., 2017; Gizachew et al., 2019). In streptococci, genes encoding resistance to TET are frequently acquired by MGEs, which also harbor erythromycin resistance determinants (Brenciani et al., 2004, 2007, 2011; Emaneini et al., 2014; Cattoir, 2016). The presence of determinants of tetracycline resistance (*tet* genes) in conjugative transposons, which can efficiently translocate among related bacteria, may explain the high prevalence of resistance (Santoro et al., 2014). There is a significant association between *tet*M and *erm*B (genetic determinant for erythromycin resistance) that has been identified among the strains of pyogenic streptococci, and it can be co-transferred among *S. agalactiae* and S. *pyogenes* strains (Brenciani et al., 2007; Emaneini et al., 2014). There is also evidence of the linkage between *tet*O and *erm*TR/*mef*A genes (Giovanetti et al., 2003) and lysogenic transfer of these genes carried by Φ m46.1 among *S. pyogenes* (Di Luca et al., 2010), that contribute to a multi-resistant phenotype.

Due to the rise of pathogens resistant to multiple antibiotics, new strategies have been proposed as an alternative to conventional antimicrobials. One such example is the use of as phage-derived lysins that degrade peptidoglycan (Maciejewska et al., 2018), which may be considered as an alternative to β-lactams, or of bacteriocins that provide a more targeted approach, i.e., strain- or species-specific (Nigam et al., 2014; Matsumoto-Nakano, 2018; Hols et al., 2019). Another emerging field of research has been the use of nanoparticles, particularly metallic nanoparticles (e.g., gold and silver), as direct antimicrobial agents, as drug delivery systems that improve the pharmacokinetics parameters (Masri et al., 2019a), or taking advantage of these nanostructures’ optical properties, e.g., photothermal ablation of cells. The potential of these new approaches against streptococci shall be further discussed in the following sections.

## Biofilms and Antimicrobial Resistance

Generally, bacteria populations may strive as planktonic, i.e., freely existing in solution, and/or sessile forming a biofilm. Biofilms are defined as tri-dimensional agglomerations of cells, attached to biotic or abiotic surfaces, and encased in a self-produced matrix composed by extracellular polymeric substances (Jamal et al., 2018). Their formation might be induced by environmental changes that cause stress cells, such as nutrient limitation and antimicrobial agents (Garrett et al., 2008; Kumar et al., 2017).

In humans, biofilms account for up to 80% of bacterial infections, according to the United States National Institutes of Health (Khatoon et al., 2018). One of the most important characteristics of biofilms is their ability to increase bacterial tolerance to antimicrobial agents. Biofilms protect the microorganism not only from antimicrobial agents but from nutrients scarcity, mechanical forces, and from the host’s immune system. Several *in vitro* studies demonstrated that bacterial biofilm could become 10 to 1,000 times more resistant to the effects of antimicrobials as their planktonic counterparts (Melchior et al., 2006). Therefore, biofilm formation should be considered as a core mechanism of resistance since it increases treatment failure and promotes persistent infection.

Biofilm growth of streptococci has been extensively investigated, but insights in the genetic origin and mechanisms of biofilm formation in this genus are limited. Although most pyogenic streptococci are able to form biofilms, there is substantial heterogeneity among strains in the strength of adherence to different surfaces. Like most bacterial genera, in streptococci biofilms, a gradient of nutrients, waste, and signaling molecules are formed, thus allowing groups of cells to adapt to different environments within the same biofilm, which may be growing at a different rate. Besides that, studies show that a biofilm-specific phenotype is stimulated in a particular subpopulation, resulting in the differential expression of mechanisms against the antimicrobials (Konto-Ghiorghi et al., 2009; Genteluci et al., 2015). Even though the resistance associated to streptococci biofilms are not entirely understood, several mechanisms have been proposed in support of increased resistance to antimicrobials. These mechanisms result from of the multicellular nature of biofilms, which leads to an additive (or synergistic) effect between the biofilm community’s protection and the conventional mechanisms of resistance referred above (Rosini and Margarit, 2015; Young et al., 2016).

Formation of biofilms also favors horizontal gene transfer between community members, thus provides conditions for the uptake of resistance genes, e.g., high cell density or accumulation of genetic elements. Some studies suggest that conjugation is more efficient in biofilms than in planktonic cells (Van Meervenne et al., 2014; Kragh et al., 2016). Marks et al. (2014) demonstrated that the biofilm microenvironment of *S. pyogenes* populations results in the induction of competence genes; therefore, it is more conducive to HGT. This study shows for the first time that *S. pyogenes* can be naturally transformed when grown as biofilms.

Overall, upon biofilm formation, there is a delayed internalization of the antimicrobial through the biofilm matrix, as the primary physical and/or chemical diffusion barrier prevents the entrance of polar and charged antibiotics. Additionally, the heterogeneous growth of the biofilm cells and activation of the stress response genes contribute to the resistance phenotype.

The extracellular polymeric substances (EPS) matrix composition is essential for the properties of the biofilm since it offers cohesion and three-dimensional architecture of biofilms (Flemming and Wingender, 2010). The EPS matrix compose 80% of the biofilm containing alginates, poly-*N*-acetyl glucosamine, extracellular teichoic acid, proteins, lipids, nucleic acids, phospholipids, polysaccharides, and extracellular DNA. EPS is 97% of water, which is found as a solvent, dictating viscosity, and mobility (Flemming and Wingender, 2010; Kumar et al., 2017; Jamal et al., 2018). For certain compounds, it is known that the EPS matrix represents an initial barrier, but recent studies showed that the biofilm matrix does not form an impermeable barrier to the diffusion of antimicrobial, and other mechanisms can contribute to promoting biofilm cell survival (Trappetti et al., 2011).

Several reports indicate that the extracellular matrix of pyogenic streptococcal biofilms is rich in proteins (Genteluci et al., 2015; Young et al., 2016; Alves-Barroco et al., 2019). In some cases, the biofilm contains a large amount of mucus-like extracellular component, probably formed by DNA released from dead cells (Alves-Barroco et al., 2019). A role for extracellular DNA was also demonstrated by the reduction of biofilms formed by SDSE isolates after treatment with DNase I (Genteluci et al., 2015). The addition of a carbohydrate oxidant, such as sodium metaperiodate, to the biofilm of SDSE indicated the presence of an exopolysaccharide, like for *Streptococcus mutans* biofilms (Liao et al., 2014) and *Streptococcus intermedius* (Nur et al., 2013). Doern et al. (2009) examined *S. pyogenes* strains from different clinical sources and demonstrated the requirement for protein and DNA in the matrix of biofilm, and only passive role for carbohydrates. This is in contrast to SDSE, for which several polysaccharides have been shown to be required (Genteluci et al., 2015).

Overall, the nature of the biofilm matrix depends on the microbial cells, their physiological status, the nutrients available, and the physical conditions. The composition of the EPS matrix likely influences the resistance against different antimicrobial classes. Responses to specific stress sources such as nutrient limitation the bacterial cell slow its growth. During biofilm development, a gradient is established, in which outer layers are metabolically active and aerobic, while and the more inner layers are anaerobic with the reduced growth rate. This slow growth has been observed in streptococci biofilms that are frequently accompanied to a significant increase in antibiotics resistance (Bjarnsholt et al., 2013; Macià et al., 2014). Several antibiotics, such as aminoglycosides, β-lactams, and fluoroquinolones, do not seem to be active in anaerobic conditions, affecting only the outermost layers of the biofilm (Borriello et al., 2004). Cell-wall active antibiotics, namely, β-lactams and glycopeptide, have minimal activity against bacteria that are not replicating and are metabolically inactive (Del Pozo, 2018).

Clinical strains response to most antibiotics is assessed according to standard MIC determination. However, several studies have indicated that, as a biofilm, the same strain/isolate may be resistant, suggesting that most of the antibiotics evaluated would be ineffective in therapy. Still, information regarding the minimum concentration for biofilm eradication of pyogenic streptococcal is scarce (Conley et al., 2003; Baldassarri et al., 2006).

Biofilm formation of *S. pyogenes* protects against some drug but does not confer complete resistance to some antibiotics, namely, penicillin and fluoroquinolone (Conley et al., 2003; Baldassarri et al., 2006; Young et al., 2016). Therapeutic failures against infections caused by *S. pyogenes* may be due to the ability to internalize human cell and biofilm formation facilitating the persistence of genetically susceptible organisms, additionally supporting the HGT, and consequently, the emergence of virulent clones (Baldassarri et al., 2006). The increased resistance of biofilms to antibiotics was also observed in SDSD and *S. agalactiae* (Mah and O’Toole, 2001; Olson et al., 2002).

As explained above, the successful treatment of infections caused by biofilm-forming bacteria is troubled due to the multidrug-resistant phenotype. Conventional antimicrobial therapy is unable to eradicate the biofilm infection. Consequently, to fight the resistance of bacterial biofilm, several different strategies and antibiofilm agents have been proposed. A promising strategy is the application of nanoparticles, which have been considered as an alternative approach to combat and biofilm-based infections (Baptista et al., 2018). Applications of nanomedicine and other alternative therapies will be discussed below.

## Alternative Antibacterial Therapies

In order to tackle the growing MDR concerns, a plethora of alternative compounds, strategies and platforms has been proposed as an alternative to conventional antimicrobials. Some of these alternatives are mere concepts whose promising *in vitro* efficacy has been the focus of attention. Many of these novel solutions have been proposed to be used alone against MDR bacteria, but many other have been proposed to be used in combinatory strategies with traditional antibacterial drugs to enhance efficacy, circumvent the onset of mechanisms of resistance.

### Bacteriocins

Bacteriocins are peptides, of prokaryotic origin, with inhibitory activity against diverse groups of microorganisms (Nigam et al., 2014; Hols et al., 2019). Several authors have documented the ability of numerous bacteriocins to inhibit the growth of pathogenic microorganisms. Here we shall refer to a general representation of bacteriocins as an alternative to traditional antibiotics. Overall, bacteriocins interact with the bacterial cell membrane and alter its properties, causing cell death. These molecules normally only target closely related species, and given their bactericidal or bacteriostatic effects, they can offer an advantage relative to conventional antibiotics since treatment could be targeted against specific pathogenic (Lopetuso et al., 2019). These peptides are typically used by commensals microbiota to colonize in the human gastrointestinal tract allowing the survival of specific communities, and thus improving gut barrier function and host immune response (Hols et al., 2019). Four major classes of bacteriocins have been identified: (i) Class I, including small heat-resistant peptides, modified post-translationally, known as “lanthionine-containing bacteriocins” (e.g., lantibiotics, sactipeptides, and glycocins); (ii) Class II, including small heat-resistant peptides (<10 kDa) post-translational modifications. These are “non-lanthionine-containing bacteriocins” which are divided into four subclasses based on their size; (iii) Class III harboring heat-labile and large proteins (>30 kDa); and (iv) Class IV including complex bacteriocins, namely, large proteins with carbohydrate and/or lipid (Pieterse and Todorov, 2010; Hols et al., 2019).

Widespread applications of bacteriocins have been documented with variable efficacy reports. There has been some experimental evidence supporting the antimicrobial properties of bacteriocin nisin (produced by *Lactococcus*) against relevant oral pathogenic bacteria. It has been shown that nisin A could inhibit the growth of cariogenic streptococci, including *Streptococcus gordonii*, *Streptococcus sanguinis*, *Streptococcus sobrinus*, and *S. mutans* (Tong et al., 2010). Additionally, it was demonstrated that the nisin associated with poly-lysine and sodium fluoride can inhibit the formation of *S. mutans* biofilms (Tong et al., 2011).

Among bacteriocins used against bovine mastitis, besides the nisin, the lacticin3147 has largely been researched. This bacteriocins has proved effective against the most mastitis-causing pathogens, namely *S. aureus*, SDSD, *S. agalactiae* and *S. uberis* (Ryan et al., 1996, 1998). Studies have shown that bacteriocins produced by several streptococci to be able to inhibit closely related strains (Nigam et al., 2014; Matsumoto-Nakano, 2018; Hols et al., 2019). Some *S. mutans* and *Streptococcus salivarius* strains that are part of the commensal microbiota of the oral cavity are also producers bacteriocin producers (Tagg, 2004; Tagg et al., 2006). Healthy microbiota of the nasopharynx also harbors bacteriocin-producing strains, including *S. salivarius* strains. The bacteriocins produced by this species have been investigated for the treatment of pharyngitis and otitis (Walls et al., 2003). In order to shield against streptococcal infections, bacteriocin-producing strains are inoculated in the nasopharynx (Walls et al., 2003). The ability of normal microbiota strains to inhibit the growth of other bacteria has a critical role in its colonization of the host and suggest that these bacteriocins provide protection against *S. pyogenes* infection (Wescombe et al., 2012).

To date, few streptococci bacteriocins against mastitis-causing pathogens have been identified. However, the natural environment of bacteriocin-producing bacteria consists of a particular field for application. *S. uberis* strains isolated from bovine mastitis bacteriocin-producing has been described, the most studied is the nisin U. This bacteriocin showed activity against important mastitis-causing pathogens, specifically *E. faecalis*, SDSD and *S. agalactiae* (Wirawan et al., 2006).

Larger bacteriocins (above 10 kDa) also produced by some streptococci strains and are identified as bacteriolytic enzymes or non-lytic inhibitory. Examples comprise streptococcin A-M57 produced by *S. pyogenes* and dysgalacticin provided by SDSE. The genes that encode for SA-M57 (*scnM57*) and dysgalacticin (*dysA*) have been found on plasmids pDN571 and pW2580, respectively (Heng et al., 2006). The DysA and ScnM57 are polypeptides with 220 and 179 amino acids, respectively, both are exported via the Sec-dependent transport pathways. Interestingly, a pW2580-like plasmid is also harbored by some *S. pyogenes* strains, emphasizing the HGT between SDSE and *S. pyogenes* (Heng et al., 2006). Overall, lateral transfer of bacteriocin production underscores the contribution of the microbial ecology within the specific niche.

Nonetheless, the broad use of bacteriocin can also confer threatening for its usage on a large scale. Usually, bacteriocin resistance is acquired by lateral transfer of the immunity gene harbored in bacteriocin-producing strain. Resistance genes located on MGE can facilitate the transfer to closely related or even different species providing the means to resist specific bacteriocins (Dicks et al., 2018).

Multidrug efflux pumps also provide resistance to bacteriocins of several bacterial species (Van Hoang et al., 2011). Furthermore, bacteriocins may be degraded by proteolytic enzymes; consequently, they may not be as stable as conventional antibiotics (Tolinački et al., 2010).

### Bacteriophage and Phage Lysins

Bacteriophages (or only phages) are viruses that specifically infect bacteria. The interaction between phage and bacteria usually involves particular receptors located in the cell membranes. Therefore, the phage is a natural killer of bacteria (Ghosh et al., 2019; Lopetuso et al., 2019). For this reason, the bacteriophages and phage proteins, namely enzymes, are extensively studied as a future alternative against bacterial infections.

There are many types of phage viruses, but the vast majority of phages can be distinguished into lytic and temperate. The most common approach for therapy involves lytic phages, which are phages that induce cell lysis, and therefore cause bacterial death (Ghosh et al., 2019), whereas the temperate phages integrate within the host genome (lysogenic conversion) (Di Luca et al., 2010). Typically, in the lytic phage life cycle, after the interaction between tail fibers and the host cell surface receptors, the phage secretes specific enzymes that degrade lipopolysaccharide, peptidoglycan and outer membrane (in Gram-negative) to inject the phage DNA. Subsequently, late genes are expressed and take control of the host cell’s to then initiate phage DNA replication. The phage DNA replicated expresses genes that encode proteins necessary for new phage particle assembly, endolysins, and holins for host cell lysis. Finally, the new phage particles are released into the environment.

The most significant factor ensuring the efficacy of phage therapy is its self-replicating nature, which distinguishes them from conventional antibiotics. Therefore, the main advantage of using phages for antibacterial treatment is that it can be administered in a low dose, that is, a small number of phages allows producing more of the particles at the infection site (Maciejewska et al., 2018).

Since their discovery in 1915 by Frederick William Twort, the phages were recognized as potential antibacterial, and due to the facility of administration and absence of side effects, phages were used immediately for antibacterial therapy (oral and topical preparations) (Maciejewska et al., 2018). The discovery and introduction of penicillin in the 1940s led to the practically total abandonment of antibacterial therapy with phage in the western countries (de Almeida and Sundberg, 2020). However, the benefits of antibiotics were lost considerably with the emergence and dissemination of bacterial resistance. The emergence of infectious diseases caused by multidrug-resistant bacterial generated an essential need for alternatives approaches to traditional antibiotics (Chang et al., 2015; Lehtinen et al., 2019). Along these lines, bacteriophages and phage-derived protein therapy get revitalized.

Since the 1980s, the phage therapy revival in western countries has been considered a possible option for combat antimicrobial resistance (de Almeida and Sundberg, 2020). Many research groups have concentrated on this theme of increasing importance, with Belgium pioneering in studies for the clinical use of phages (Pirnay et al., 2018; Jault et al., 2019). Despite the large potential of phages for antibacterial therapy, a small number of clinical trials have been performed in human patients. Besides that, few clinical trials are accepted by public health authorities, for example, the European Medicines Agency (EMA) and the Food and Drug Administration (FDA) (Rios et al., 2016). In the United States and European Union, the phages and phage-encoded enzymes classified as human therapeutic material are subjected to the same implementation regulations as traditional antibiotics (Maciejewska et al., 2018).

There has been a growing interest in phage-derived enzymes with antibacterial activity, including lysins (degrade peptidoglycan), and depolymerases (that degrade polysaccharide, e.g., capsule, biofilm matrix, and lipopolysaccharide) (Maciejewska et al., 2018). Regarding the application of these enzymes, previous studies, including animal models and clinical trials, showed antibacterial activity and reaffirmed the safety of its use (Lopetuso et al., 2019). Nonetheless, the current legislation limits the use of recombinant enzymes in human therapy, mainly for systemic therapy (Schmelcher et al., 2012).

The potential of phage against biofilm-forming bacteria has been demonstrated. The ability of the bacteria to produce biofilms has been considered the most common reason for failure therapeutic of antibiotics due to the impermeability of the biofilm matrix and the diversity of bacterial cells at different metabolic stages. Studies show that some phages have naturally depolymerases able to degrade the biofilm matrix (Abedon, 2015b). Probably, the depolymerases have evolved in response to polysaccharide of the biofilm matrix that covers the membrane receptor required for the interaction between the phage particle and the host cell and the subsequent attachment. The phages can also infect metabolically inactive bacteria of the biofilm since the receptor is present, but the lytic cycle stays pendent until bacterial metabolism to be active (Pearl et al., 2008). The complete eradication by one phage is rather difficult due to a complex structure of the mature biofilm. A combined action (combined therapy) has been suggested as a valid approach, in which depolymerase that degrades polysaccharides of the matrix allowing the phage or antibiotics to achieve the bacteria (Abedon, 2015a). Phage lysins have been also effectively used to remove bacterial biofilms (Meng et al., 2011; Shen et al., 2013; Rico-Lastres et al., 2015).

Bacteriophages infection occurs through specific protein receptors on the bacterial surface, which is the cause of extreme selectivity of these agents but also their main limitation. Due to this high specificity, phage therapy is of narrow-spectrum compared to traditional antibiotics whose targets are general pathways and processes common to most bacteria (e.g., protein synthesis). Unlike traditional antibiotics, one particular phage has a restricted number of strains as target, in other words, several and different phages are required to combat one only bacterial species. Moreover, phage-based therapy requires the previous identification of bacteria, causing infection to the isolation of specific lytic phage. Methods to isolate bacteriophages with broad-host-range and modifications to expand the specificity have been the target of several approaches, which could reduce the number of phages needed per species (Fairhead et al., 2017; Hyman, 2019). Moreover, the use of a “phage-cocktail” (composed of strictly lytic phages) can expand the spectrum of action and be administered in combination with other antibacterial agents, thus, increasing the potential of phage therapy.

The success of antibacterial phage-based therapy broadly depends on the patient’s immune system (PIS) that may recognize and inactivate viral particles. A low-level of antibodies specific to several viral proteins can naturally exist. Moreover, during phage therapy, the activation mechanisms of the immune response can be triggered; thus, phage may be recognized by the PIS, severely compromising the therapeutic effectiveness (Borysowski et al., 2012).

The activity of antibodies in phage lysines inactivation has also been investigated. Studies have shown that antibodies were effective in reducing the half-life of these enzymes (Rashel et al., 2007; Jun et al., 2014). Nonetheless, modification of lysins by dimerization to broaden their half-life has been investigated (Resch et al., 2011). For example, the ClyS (chimeric endolysin) that showed insensitive to antibodies (Pastagia et al., 2011).

Furthermore, phage particles may undergo denaturation by conformational changes irreversible or reversible. A proposed solution is encapsulation within nanocarriers to become the particles insoluble and protecting them from the digestive and immune system (Balcão et al., 2014; Rios et al., 2018). Another critical question is to get the phage particles to the infection site, given that phages do not have pharmacokinetic properties (Ghosh et al., 2019). One of the biggest concerns of phage-based therapy is the gap in understanding of phage-bacteria-human interaction, namely their safety. Concerning phage therapy in immunocompromised patients, although considered safe, its use may be less effective with more associated risks (Roach et al., 2017).

Another aspect associated with security difficulties is horizontal gene transfer. Although low, there is the possibility of HGT affect the pathogenic potential of co-existing bacterial strains by sharing of antibiotic resistance and virulence genes into the population (Lim et al., 2014). The proper phage selection against a given infection still is a challenging question. Moreover, clinical phage resistance *in vivo* also is a complicated issue.

Recent studies in animal models suggest that bacterial mutations resulting in phage-resistance may enhance the pathogen’s fitness in its regular niche within the host (Oechslin, 2018). Experimental data showed that phage-resistance occurs in 80% of researches targeting the intestinal environment and 50% of investigations with a model of sepsis. In human studies, phage-resistance has also been observed.

In the pyogenic streptococcus group, strains can escape to phage attack through several mechanisms, comprising spontaneous mutations of the genes encoding receptor, restriction-modification systems, abortive infection mechanisms, and adaptive immunity mediated by CRISPR-Cas systems (Almeida et al., 2016; Euler et al., 2016). The spontaneous mutation is the principal mechanism emergence of resistance and phage–bacterial coevolution. The mutations may provide resistance by changing the bacterial surface molecules in particular phage receptors, and that also determine phage specificity.

Concerning modification-restriction mechanisms, it is based on its abilities to restrict incoming foreign genetic material and to protect host DNA from restriction through modification (methylation, for example) of specific bases in the DNA sequence. Due to host DNA modification, unmodified sequences are then assumed to be foreign and thus cleaved by restriction endonuclease (Stern and Sorek, 2011). Usually, this mechanism causes the death of phage particles but preserves the host. If the system fails, intruding phages will be replicated and modified by the cell, becoming resistant to restriction. In abortive infection (Abi), the host mechanisms arrest phage development at its different steps, e.g., phage transcription, genome replication, or phage genome assembly. Abi mediated resistance ultimately results in the death of both the bacteriophage and the host. It is a selfless defense mechanism since the host dies, but the surrounding population is benefitted (Stern and Sorek, 2011). Although some of these systems work similarly to toxin-antitoxin systems, Abi systems are vastly diverse, and their modes of action are still not completely understood.

The CRISPR-Cas system consists of a multistep process by which small fragments of foreign nucleic acids (or protospacers) are first recognized and included in the host genome. Afterward, these fragments (or spacers), along with Cas proteins, are used as an adaptive immune system that recognizes, degrades or silences foreign nucleic acids (Bondy-Denomy et al., 2013; Rath et al., 2015). However, phages have acquired mutation-based strategies to evade CRISPR/Cas systems, e.g., losing their spacer sequences or encoding products that target Cas proteins (Stern and Sorek, 2011).

### Nanoparticles

**FIGURE 3:**
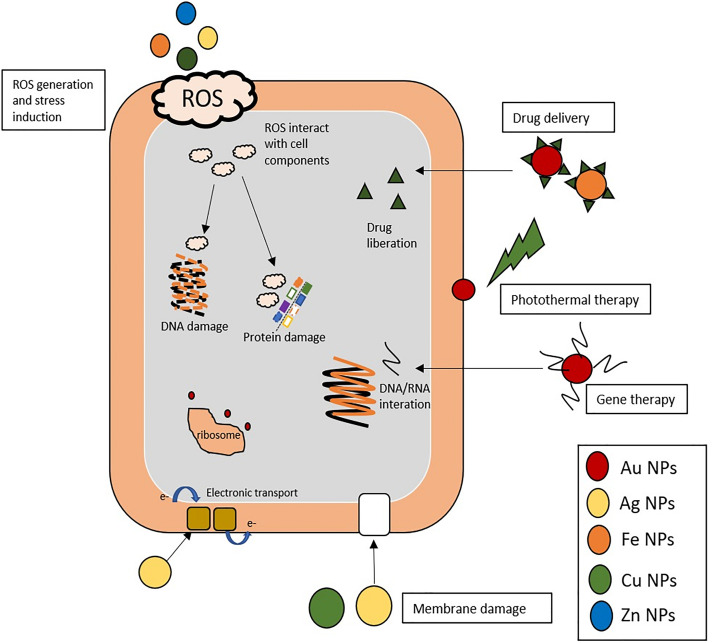
Different mechanism of action of metallic NPs in bacteria. Nanoparticles induce wide effects in bacterial metabolism by different approaches: (i) ROS generation: Ag, Fe, Cu, and Zn NPs induce ROS (reactive oxygen species), the ROS generated are highly reactive toward biological molecules such as proteins and DNA and interact and damage them. (ii) Damage membrane: Ag and Cu NPs interact with chemical groups of bacterial membrane (sulfate or phosphate) and disturb the normal functions. (iii) Drug and gene delivery systems: Au and Fe NPs could be carrier of gene moieties (DNA/RNA) that interact with bacterial gene, or deliver drug improving some pharmacodynamic parameters. (iv) Ribosome: Au NPs inhibit the union of transfer RNA (tRNA) to ribosome. (v) Photothermal therapy of Au NPs mediated by laser irradiation that disturb the membrane structure. (vi) Bacterial respiration: Ag NPs alter the electronic transport and inhibit the respiratory chain.

Nanomaterials have recently gained great interest due to the variety of applications in biomedicine (McNamara and Tofail, 2017). Nanomaterials comprise a range of constructs, materials and functional systems of particles whose size is between 1 and 100 nm. Particularly, these nanotechnology-based materials have found plenty of applications as alternative tools to traditional antibiotics and, more interestingly, as means to prevent the surge in antibiotic resistance (Baptista et al., 2018). The use of nanoparticles as antibiotic therapy has relied on these nanostructures acting as carrier of drugs, either via integration or incorporation into the nanoformulation, or adsorbed to the surface so as to improve biodistribution and pharmacokinetics, e.g., solubility, controlled drug liberation and therapeutic effectivity (Gao et al., 2018), the main mechanism of action of metallic nanoparticles in bacteria are described in Figure 3. However, several nanomaterials have been proposed as antimicrobials, with particular emphasis metallic nanoparticles (the most recent application of these metallic nanoparticles against *Streptococcus* spp. are shown in Table 3).

**TABLE 3:** Application of metallic nanoparticles against *Streptococcus* spp.

Metal	Synthesis method		Bacteria	Highlights	References
Silver	Green synthesis	*Terminalia mantaly* extract	*S. pneumoniae*	The biogenic *Terminalia mantaly*-Ag NPs showed significant antibacterial activity compared to the respective extracts	Majoumouo et al., 2019
		*Allium cepa* and *Allium sativa* extract	*S. pneumoniae*	AgNPs exhibited antibacterial activity against selected vaginal bacteria	Bouqellah et al., 2019
		Fruit extract of *Prosopis farcta*	*S. pneumoniae*	AgNPs increased the antioxidant and antibacterial activity compared with the extract alone, due to high content in phenolic compounds.	Salari et al., 2019
		*Tapinoma simrothi*	*S. pyogenes*	AgNPs with effective antimicrobial activity in a wide range of bacteria	Sholkamy et al., 2019
	Chemical synthesis	Silver nitrate reduced by sodium borohydrate	*S. pyogenes*	AgNPs as carrier of new quinazolinone compounds showed enhanced antibacterial activity	Masri et al., 2019a
Gold	Green synthesis	*Justicia glauca* extract	*S. mutans*	AuNPs coated with antibiotic increased efficacy against a broad range of bacteria	Emmanuel et al., 2017
		Resveratrol as a green reducing agent	*S. pneumoniae S. pyogenes*	AuNPs-resveratrol increased efficacy against *S. pneumoniae* compared to resveratrol	Park et al., 2016
	Chemical synthesis	Reduction of gold (III) chloride trihydrate by sodium citrate	*S. pneumoniae*	Uptake of AuNPs by *S. pneumoniae* associated the antibacterial activity to the formation of inclusion body of AuNP (IB-AuNPs), composed by proteins, carbohydrates and lipids. Some proteins associated with IB-AuNPs could be used for new strategies	Ortiz-Benítez et al., 2019
		Citrate reduction of gold (III) chloride trihydrate	*S. mutans*	Combination of AuNPs and diode irradiation decreased CFUs	Sadony and Abozaid, 2020
Gold-silver	Gold-silver nanocages via galvanic replacement reaction		*S. mutans*	Au-Ag nanocages promoted the inhibition of *S. mutans*	Wang et al., 2016
Gold-titanium	Commercial TiO_2_ nanotubes with Au via direct current plasma sputter		*S. mutans*	Ti nanotubes sputtered with Au nanorod irradiation increased the inhibitory effect against *S. mutans*	Moon et al., 2018
	*Green synthesis: Terminalia chebula* bark extract		*S. pneumonia*	Au-TiNPs loaded with carbon nanotubes and irradiated under visible light showed higher antimicrobial activity than ampicillin	Karthika and Arumugam, 2017
Zinc-silver	Polymeric precursor and coprecipitation		*S. mutans*	Zn-AgNPs inhibit *S. mutans* biofilm formation (dentistry)	Dias et al., 2019
Iron	Green synthesis	*Agrewia optiva* and *Prunus persica* extracts	*S. mutans S. pyogenes*	FeNPs provided antimicrobial activity and antioxidant capacity associated to compounds from extracts	Mirza et al., 2018
	Chemical synthesis	Commercial NPs	*S. mutans*	Chitosan coated FeNPs as carrier for chlorhexidine; bacteria eradication and antibiofilm effect	Vieira et al., 2019
		Ferric chloride and ferrous chloride tetrahydrate	*S. mutans*	FeNPs on surface for eradication of *S. mutans*	Javanbakht et al., 2016
		Solvothermy employing iron (III) chloride	*S. mutans*	Vitamin B_2_ coated FeNPs promoted antibacterial activity	Gu et al., 2020
Copper	Chemical methods: copper acetate as precursor		*S. mutans*	Hybrid Cu-chitosan NPs reduced MIC and minimum bactericidal concentration	Covarrubias et al., 2018
	Commercial NPs		*S. mutans*	CuNPs added to orthodontic composite inhibited the growth of *S. mutans*	Toodehzaeim et al., 2018
Zinc	Zinc acetate dihydrate as precursor		*S. pneumoniae*	ZnNPs reduced IMC and showed anti-biofilm formation activity	Bhattacharyya et al., 2018
	Green synthesis: *Costus igneus* extract as capping and reducing agent		*S. mutans*	ZnNPs showed a dose dependent antibacterial and antibiofilm effect against *S. mutans*	Vinotha et al., 2019

Some of the value of nanotechnology for antimicrobial therapy relate to modulation of the pharmacokinetic profile, where nanoparticle mediated drug delivery might improve conveyance of the drug to the desired tissue. Furthermore, nanoparticles could be designed to enhance solubility, control the release of drug and increase clearance from the organism, thus improving the therapeutic window of the cargo drug. Also, some synergistic strategies may be used, such as photothermal ablation of cells, where combination to the “traditional” chemotherapeutic may lead to an increase of efficacy. Nevertheless, there are some limitations to the use of these nanomaterials before their successful translation to clinics. One is the limited amount of data on the use of such systems to tackle infection in *in vivo* models, thus preventing adequate assessment of optimal dose, appropriate administration routes and possible interaction of nanoparticles with cells and tissues, whose toxicological profile is not completely understood (Baptista et al., 2018; Lee et al., 2019).

#### Silver Nanoparticles

Traditionally, Silver (Ag) has been employed as antimicrobial, namely silver sulfadiazine and silver nitrate (Lansdown, 2006), which relied on the release of Ag+ that triggers a range of processes resulting in hampered bacterial growth. Silver nanoparticles has allowed improved release of silver ions and, thus, enhance the bactericide action (Dakal et al., 2016). Silver nanoparticles (AgNPs) may be synthesized by different protocols relying on thermic vaporization, chemical or photochemical reduction of silver ions to form nanoparticles that are then capped either by the same reagent or by additional compounds, which promote solubility and stability (Lee and Jun, 2019). The most used method is based on using citrate as reducing and capping agent of silver salts. Nowadays, other methodologies using biological extracts (e.g., plant extracts) has been employed for the “green synthesis” of this nanomaterial (Masooleh et al., 2019). Regardless of the synthesis method, the antibacterial activity of AgNPs depends on the size and shape of the particles; for example, the antibiotic effect increases with smaller sizes due to the dramatic increase in surface area available for ion release and/or to interact with the bacteria. In fact, some authors propose that AgNPs between 1 and 10 nm could interact more efficiently with the bacteria cell membrane (Morones et al., 2005), and spherical nanoparticles seem to be more effective in bacterial eradication than triangles or cylinders (Raza et al., 2016). Still, for AgNPs, size is the stronger determinant associated with antibiotic activity, which is clear in conceptual studies of efficiency for *Pseudomonas aeruginosa* and *Escherichia coli* eradication (Raza et al., 2016). However, AgNPs have shown biocidal effects against a range of bacterial species with clinical interest, such as *Staphylococcus epidermidis*, *Enterococcus faecalis*, *Vibrio cholerae*, and *Salmonella* spp. (Morones et al., 2005; Sánchez-López et al., 2020).

Although the biocidal action of AgNPs has been attributed mainly to the Ag+ release, the actual mechanism is not yet completely elucidated. Crucial to their effect is the fact that AgNPs tend to accumulate at the membrane where they progressively aggregate, allowing silver ions to interact with different functional groups, such as sulfate or phosphate, and disturb the function of the bacterial membrane, promoting its rupture and liberate the cytoplasmatic content (Le Ouay and Stellacci, 2015). Other studies have suggested that Ag+ is able to interact and inactivate some biological structures and affect the bacteria’ respiratory process, namely inhibiting the respiratory chain (Costa et al., 2010). However, perhaps the most widely accepted hypothesis is the production of reactive oxygen species (ROS), like superoxide or hydrogen peroxide, which interact with the lipids, proteins or DNA, promoting alteration in the normal functions, triggering lysis and cell death (Quinteros et al., 2016).

AgNPs have also found a range of industrial applications that require some level of inhibition of bacterial growth. One such examples is the development of new tools for odontology, where AgNPs have been added to customary compounds for dental implants (e.g., polydopamine and titanium) to improve biocompatibility and provide for added antibacterial efficacy against *S. mutans*, commonly implicated in the caries disease (Choi et al., 2019). Green synthesized AgNPs by *Epigallocatechin gallate* (green tea extract) as reducing and chitosan as capping agent decreased the MIC and MBC against *S. mutans* (Yin et al., 2019). What is more, these AgNPs induced lower amounts of lactic acid and polysaccharides in the biofilm, thus enhancing the protective action of the nanoparticle extracts. The synergistic effect of the bio-extracts and AgNPs may be associated to the bactericide activity of the green tea polyphenols and the large surface area of AgNPs which increase the contact with bacteria and facilitate disruption of cell metabolism. In addition, *E. gallate* is able to inhibit the *S. mutans* glucosyltransferase reducing bacterial adherence and biofilm formation. Another interesting application for AgNPs has been its inclusion in toothpaste formulations with promising antibacterial efficacy against *S. mutans* (Ahmed et al., 2019).

Furthermore, the synergistic effect with other conventional antibiotics makes possible the application of AgNPs as an alternative tool to tackle MDR strains. In fact, AgNPs and conventional antibiotics exert their biocidal action via different mechanisms and, therefore, their combination would prevent the development of added resistance. For example, clindamycin has already been combined with AgNPs resulting in lower MICs in a synergistic effect and rifampicin coupled to AgNP increased the antibiotic effect against methicillin resistant bacteria (Khan et al., 2019a).

#### Gold Nanoparticles

Gold nanoparticles (AuNPs) have also been employed in different fields of biomedical research due to their ease of synthesis, biocompatibility and low toxicity to higher eukaryotes. They are easier to functionalize with different biological moieties like DNA, mRNA, peptides, etc. than their silver counterparts. Moreover, AuNPs present remarkable optical and photoelectric properties that have demonstrated high potential toward the development of new therapy tools (Amendoeira et al., 2020). The chemical processes for the synthesis of AuNPs are similar to those of AgNPs, where the citrate reduction method is clearly the most used method. Still, in the last years, as for AgNPs, several green synthesis methods with plant or other extract have been proposed (Khan et al., 2019b).

AuNPs have been reported to exhibit antimicrobial activity against a wide range of bacteria and fungus (Tao, 2018). Several mechanisms of action have been highlighted as the basis of their antimicrobial properties, namely: AuNPs may bind to the membrane of bacteria, modify the membrane’s potential, decrease intracellular ATP levels, disturb intracellular trafficking, aggregate together with proteins and disturb the assembly of tRNA to the ribosome (Cui et al., 2012). Perhaps, the main aspect related to antimicrobial efficacy relates to nanoparticle dispersion and the AuNPs’ surface roughness that could interact with the bacteria membrane (Lima et al., 2013). AuNPs have also been proposed as drug carriers, conveyors of gene therapy and photothermal therapy (Li et al., 2019; Masri et al., 2019b).

The use of AuNPs as drug delivery systems for traditional antibiotics has made possible to administrate drugs more effectively and uniformly distributed toward the target tissue, improving the efficacy and biocompatibility of antibiotic-conjugated AuNPs. The surface of AuNPs may be easily functionalized with small ligands harboring carboxylic acid, hydroxyl, or amine functional groups that can then be used to conjugate antimicrobials (Lee et al., 2017). The so assemble nanoformulation improves solubility of non-water-soluble drugs and allows for controlled and localized release of the antibiotic, for example with an external stimulus (Canaparo et al., 2019). For example, a formulation of imipenem and meropenem on AuNPs increased the antibacterial effect against carbapenems resistant Gram-negative bacteria, like *Klebsiella pneumoniae*, *Proteus mirabilis* and *Acinetobacter baumannii* isolated from human patients, and decreased the MIC while potentiating the effect in antibiotic kill test (Shaker and Shaaban, 2017). Moreover, these studies showed a size-dependent efficacy of the drug system, with optimal efficacy for 35 nm nanoparticles. Another advantage antibiotic delivery system mediated by AuNPs is the drug-controlled release, where AuNPs loaded with Amphotericin B showed an increase of biocidal efficiency of 78%, with less cytotoxicity and hemolytic toxicity to the host when compared to the antibiotic alone (Kumar et al., 2019).

When AuNPs are irradiated by light with an appropriate wavelength, they tend to convert the received energy into heat, thus resulting in a hyperthermal effect capable to induce damage to the membrane structure (Kirui et al., 2019; Amendoeira et al., 2020). The antimicrobial effect through the use of photothermally active nanomaterials may become an interesting tool against antibiotic resistance. For example, near-infrared (NIR) radiation is useful to promote hyperthermy based on AuNPs, effective against *S. aureus* and *E. coli* after 10 min of irradiation at 808 nm (Alhmoud et al., 2017). NIR photothermy using AuNPs has been also used as an efficient technique to eliminate a broader range of microorganism with improved antibiofilm activity. In fact this approach was demonstrated effective at damaging the cell wall of streptococci, such as *S. mutans*, *S. sobrinus*, *Streptococcus oralis* and *S. salivarius* (Castillo-Martínez et al., 2015).

The combination of these two metals, Silver and Gold, in alloy nanoparticles has also been proposed as suitable nanoagent against microbes. In fact, such approach combines the improved stability and ease of functionalization provided by gold, with the higher antimicrobial activity of silver, while avoiding some problems associated with the aggregation and toxicity to the host (Dos Santos et al., 2012). The positive results of this association have been proposed in a system where gold-silver alloy “nanoflowers” decreased the MIC against *E. coli* three-fold when compared to AgNP alone (Yan et al., 2018). Gold-silver alloy nanoparticles have shown their potential to eradicate biofilm and reduce the MICs against Gram-positive and Gram-negative bacteria, which could then be used to circumvent drug resistance (Ramasamy et al., 2016). Kyaw et al. (2017) showed that submitting triangular AuNPs coated by silver to laser irradiation, induced a change of shape to spherical and increased the antibacterial activity.

#### Other Metallic Nanoparticles

Nanoparticles employing iron (Fe) have been applied due to their antimicrobial properties, which has been associated with the generation of ROS (Arakha et al., 2015). The most used is iron oxide nanoparticles which provide good efficiency in a wide range spectrum mediated by the damage in different structures like proteins or DNA (Saqib et al., 2019). Most of these iron nanoparticles present some magnetic properties that may be used for a range of applications, from diagnostics to therapeutics (Rodrigues et al., 2019). Magnetic nanoparticles have been shown to interfere with the thiol groups at the respiratory base of bacteria and, thus, assisting in disrupting effective metabolism, resulting in biocidal activity against some drug resistant bacteria (Madubuonu et al., 2019). Ion NPs have also been used as delivery vehicles for antibiotics, such as chlorhexidine and erythromycin against *S. mutans* (Vieira et al., 2019).

Copper nanoparticles have also been used as antimicrobial against a wide range of microorganisms including bacteria, fungi, and even algae (Hou et al., 2018; Sardella et al., 2018). As for AgNPs, the size is related to CuNP activity due to the dramatic increase of the surface/volume ratio, which promote the generation of ROS that trigger cell damage according, such as oxidation of proteins, cleavage of DNA/RNA molecules or lipid peroxidation in membranes (Shaikh et al., 2019). Usually, CuNPs are combined within polymers or functionalized in core-shell structures to provide stability and control possible ion leakage (Anyaogu et al., 2008). For example, CuNPs coated with chitosan showed an antibacterial effect comparable to that of traditional oral antimicrobial agents (chlorhexidine and cetylpyridinium chloride) against *S. mutans* (Covarrubias et al., 2018). In another example, the combination of AgNPs and CuNPs was shown to have a preventive and therapeutic effect in mastitis caused by *S. agalactiae* (Kalińska et al., 2019).

Some authors have studied the antibacterial activity of zinc NPs (ZnNPs) against *Streptococcus mitis*, where the biocidal action was associated to ROS induction identified via the increase of superoxide dismutase activity (SOD) (Khan et al., 2016). Moreover, ZnNPs showed the capability to inhibit the formation of biofilm by *S. mitis* in a dose dependent manner, corroborated the evaluation of bapA1 gene expression, which is associated to generation of the biofilm. In another study, ZnNPs showed the capability to inhibit *S. sobrinus* biofilm formation (Aydin Sevinç and Hanley, 2010).

## Concluding Remarks

The progressive emergence of resistance to conventional antibiotics is reducing the ability to control infectious diseases and, particularly, those caused by pyogenic streptococci. To combat this public health threat, several alternative strategies have been proposed, and the promising efficacy *in vitro* of some of these antibacterial approaches has been the focus of attention.

Despite the progress achieved to date, most alternative approaches are of narrow spectrum unlike the broad-spectrum of conventional antibiotics. However, the combined action of one of these alternative approaches with traditional antibiotics may increase the success rate of therapeutics once that most new strategies attenuate bacterial pathogenesis allowing bacteria to be eliminated by antibiotics and action of the immune system. Moreover, combination therapies may decrease the selective pressure for resistance to antibiotics, and consequently to reduce the rate of emergence of resistance.

Bacteriocins are considered as a hopeful therapeutic alternative caused by its proven efficacy and chemical structural and functional diversity. Nonetheless, the broad use of bacteriocin can also confer threatening for its usage on a large scale, namely, the possible resistance to bacteriocins could limit their future way. Bacteriophages allow the development of specific therapies, phage-derived enzymes can be used as a substitute for conventional antibiotics, for example, phage-derived lysins that degrade peptidoglycan can be considered as an alternative to β-lactam antibiotics. However, the advancement of new laws that regulate the use of Bacteriophages and phage-derived enzymes is necessary. The application of nanomaterials may provide for new therapy tools to assist in tackling the traditional mechanisms of resistance. Still, there is plenty of work ahead to facilitate the translation to the clinics, namely toward better characterization of these materials, the capability to effectively scale-up for widespread use, and clarification of toxicity aspect, which altogether pave the way for robust assessment in clinical trials. Nowadays, the cost associated with the development of nanotechnology platforms is high and, consequently, the use of more conventional therapies are still preferred.

The most significant concern safety of alternative therapies is the gap of understanding of interaction with the human host. Thus, evaluate the impacts of alternative therapies on the host is essential for future widespread application. Future studies must investigate *in vivo* efficacy of combination therapy to assess their potential, impact of the host, and evolution of bacterial resistance.

## Author Contributions

CA-B and LR-G: revision of literature and drafting the manuscript. AF and PB: revision of literature and drafting and editing the final manuscript. All authors contributed to the article and approved the submitted version.

## Conflict of Interest

The authors declare that the research was conducted in the absence of any commercial or financial relationships that could be construed as a potential conflict of interest.
